# Pressure-Induced Phase Transition and Band Gap Decrease in Semiconducting β-Cu_2_V_2_O_7_

**DOI:** 10.1021/acs.inorgchem.1c03878

**Published:** 2022-02-14

**Authors:** Robin Turnbull, Javier González-Platas, Fernando Rodríguez, Akun Liang, Catalin Popescu, Zhangzhen He, David Santamaría-Pérez, Plácida Rodríguez-Hernández, Alfonso Muñoz, Daniel Errandonea

**Affiliations:** † Departamento de Física Aplicada - Instituto de Ciencia de Materiales, MALTA Consolider Team, Universidad de Valencia, Edificio de Investigación, C/Dr. Moliner 50, Burjassot, 46100 Valencia, Spain; ‡ Departamento de Física - Instituto Universitario de Estudios Avanzados en Física Atómica, Molecular y Fotónica (IUDEA), MALTA Consolider Team, Universidad de La Laguna, Avenida Astrofísico Fco. Sánchez s/n, La Laguna, Tenerife E-38204, Spain; § MALTA Consolider Team, Departamento de Ciencias de la Tierra y Física de la Materia Condensada, Facultad de Ciencias, Universidad de Cantabria, 39005 Santander, Spain; ∥ CELLS-ALBA Synchrotron Light Facility, 08290 Cerdanyola del Vallès, Barcelona, Spain; ⊥ State Key Laboratory of Structural Chemistry, Fujian Institute of Research on the Structure of Matter, Chinese Academy of Sciences, Fuzhou, Fujian 350002, China; # Departamento de Física, Instituto de Materiales y Nanotecnología, MALTA Consolider Team, Universidad de La Laguna, La Laguna, E-38204 Tenerife, Spain

## Abstract

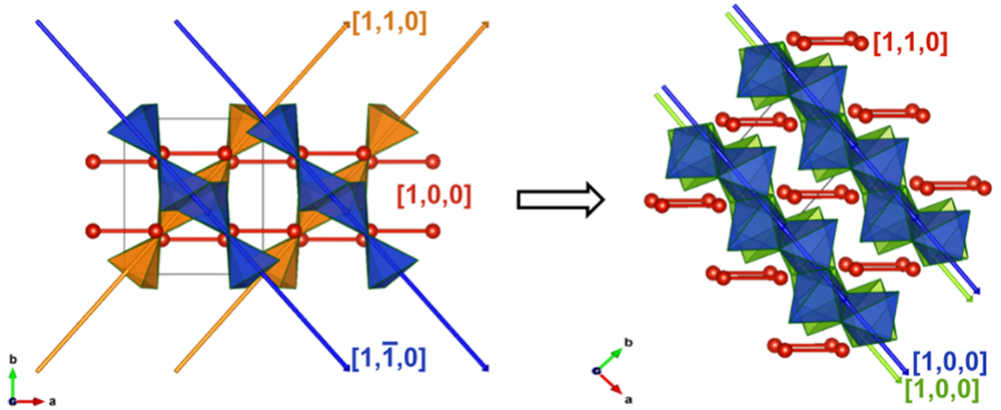

The understanding of the interplay between crystal structure and electronic structure in semiconductor materials is of great importance due to their potential technological applications. Pressure is an ideal external control parameter to tune the crystal structures of semiconductor materials in order to investigate their emergent piezo-electrical and optical properties. Accordingly, we investigate here the high-pressure behavior of the semiconducting antiferromagnetic material β-Cu_2_V_2_O_7_, finding it undergoes a pressure-induced phase transition to γ-Cu_2_V_2_O_7_ below 4000 atm. The pressure-induced structural and electronic evolutions are investigated by single-crystal X-ray diffraction, absorption spectroscopy and *ab initio* density functional theory calculations. β-Cu_2_V_2_O_7_ has previously been suggested as a promising photocatalyst for water splitting. Now, these new results suggest that β-Cu_2_V_2_O_7_ could also be of interest with regards to barocaloric effects, due to the low phase -transition pressure, in particular because it is a multiferroic material. Moreover, the phase transition involves an electronic band gap decrease of approximately 0.2 eV (from 1.93 to 1.75 eV) and a large structural volume collapse of approximately 7%.

## Abstract 2

The semiconducting antiferromagnetic material β-Cu_2_V_2_O_7_ is found to exhibit a pressure-induced phase transition to γ-Cu_2_V_2_O_7_ below 4000 atm. The pressure-induced structural and electronic evolutions are investigated by single-crystal X-ray diffraction, absorption spectroscopy, and *ab initio* density functional theory calculations. The phase transition is characterized by an electronic band gap decrease of approximately 0.2 eV (from 1.93 to 1.75 eV) and a large structural volume collapse of approximately 7%.

## 1 Introduction

### 1.1 Introduction

Copper vanadate materials constitute an important class of semiconducting inorganic compounds which exhibit a wide variety of chemical compositions: CuV_2_O_6_, Cu_2_V_2_O_7_, Cu_3_V_2_O_8_, Cu_4_V_2_O_9_, and Cu_5_V_2_O_10_. Of these, copper(II) pyrovanadate, Cu_2_V_2_O_7_, has been highlighted as a favorable material for the photocatalytic splitting of water into hydrogen and oxygen due to its band gap energy.^1−3^ Therefore, copper vanadate materials may play an essential role in the global shift toward renewable energy sources. Cu_2_V_2_O_7_ exhibits three known polymorphs, known as α, β, and γ, all of which are formed at elevated temperatures, either naturally occurring (α and β) or synthesized in the laboratory (γ), and all of which are (meta)stable at ambient conditions.^4−9^

The present work investigates β-Cu_2_V_2_O_7_ under high-pressure conditions at ambient temperature to explore the effect of pressure on the crystal and electronic structures. β-Cu_2_V_2_O_7_ has a number of interesting and useful properties; for example, β-Cu_2_V_2_O_7_ is a semiconducting antiferromagnetic material which has an (indirect) electronic band gap energy of ∼2 eV, which is optimal for absorbing energy within the solar range.^10^ β-Cu_2_V_2_O_7_ also exhibits interesting magnetic properties, due to its spin-1/2 honeycomb lattice of Cu^2+^ [3d^9^] ions, including quasi-1D antiferromagnetism.^11−13^ Finally, β-Cu_2_V_2_O_7_ is also known to exhibit a negative thermal expansion at ambient pressure.^14,15^ None of the Cu_2_V_2_O_7_ phases has previously been studied at high pressure; therefore, an additional motivation for this high-pressure investigation is to study mechanical similarities between the negative thermal expansion and pressure-induced volume decrease.

Herein, we report an experimental high-pressure single-crystal synchrotron X-ray diffraction (XRD) study of β-Cu_2_V_2_O_7_ under compression up to 4 GPa at ambient temperature. We present unambiguous evidence of a pressure-induced first-order phase transition from the monoclinic β-phase to the triclinic γ-phase between 0.14 and 0.40 GPa. We also investigate the electronic structure via absorption spectroscopy and *ab initio* density functional theory calculations, in particular the optical band gap and sub-band d–d transitions associated with Cu^2+^ coordination complexes, finding the phase transition to be characterized by a 0.2 eV band gap decrease and a decrease in the average crystal field strength. We also report a detailed study of the pressure evolution of both of the crystal structures, associated crystallographic parameters and physical properties, including the bulk moduli (via a pressure–volume equation of state (EoS)) and isothermal compressibility tensors of both β- and γ-phases.

## 2 Methods

### 2.1 Sample Preparation

Single crystals of β-Cu_2_V_2_O_7_ were grown by a flux method using SrV_2_O_6_ as a flux according to ref (16). A mixture of high-purity CuO, V_2_O_5_, and SrCO_3_ was ground fully and evenly with ethanol (99%) in an agate mortar. The mixture was packed into a platinum crucible (40 × 40 × 45 mm^3^) which was then placed in a homemade electric furnace. The furnace was heated up to 950 °C and kept at this peak temperature for 20 h. The furnace was then cooled slowly to 750 °C at a rate of 0.5 °C/h. The furnace was finally cooled down to room temperature at a rate of 100 °C/h. With the above growth procedure, single crystals of β-Cu_2_V_2_O_7_ with a size of 3 × 3 × 5 mm^3^ were obtained by mechanical separation from the crucible. Alternative synthesis methods for β-Cu_2_V_2_O_7_ also exist.^2,17^

### 2.2 Measurements

Angle-dispersive single-crystal XRD data were acquired in two ways. First, data were acquired at ALBA Synchrotron^18^ (Barcelona, Spain) on the BL04 - MSPD beamline using a monochromatic beam λ = 0.4246 Å focused to a spot size of 20 × 20 μm^2^. A SX165 Rayonix Mar CCD detector was used to record the data. Second, single-crystal XRD data were collected in-house at the University of La Laguna. CrysAlisPro^19^ was used to collect, index, scale, and apply numerical absorption corrections to the data. Single-crystal diffraction measurements (SC-XRD) were carried out at room temperature using a Rigaku SuperNOVA diffractometer equipped with an EOS CCD detector and a Mo radiation microsource (λ = 0.71073 Å). All measurements were processed with the CrysAlis software version 1.171.40.71.^20^ Numerical absorption correction based on Gaussian integration over a multifaceted crystal model was applied using the ABSORB7 program.^21^ For HP measurements we used a Mini-Bragg DAC from Almax-EasyLab, with an opening angle of 85° and anvil culets of 500 μm diameter, fitted with a stainless-steel gasket containing a hole of 200 μm diameter and 75 μm depth. A 4:1 methanol–ethanol mixture (ME) was used as a pressure-transmitting medium.^22^ The sample was placed on one of the diamonds anvils (diffracted side) together with a small ruby sphere as a pressure sensor.^23^ The structure was refined, for each pressure, using previous results as starting points, on *F*^2^ by full-matrix least-squares refinement using the SHELXL program.^24^

The optical absorption spectra were acquired using the sample-in sample-out method on the in-house optical setup at the University of Valencia, consisting of a visible–near-IR spectrometer (Ocean Optics Maya2000 Pro), a tungsten filament lamp, fused silica lenses, and reflecting optical objectives. The intensity of the light transmitted through the sample (*I*(ω)) was normalized against the intensity of the light transmitted through the 16:3:1 methanol–ethanol–water PTM (*I*_0_(ω)). Single crystals of β-Cu_2_V_2_O_7_, approximately 100 × 100 × 40 μm^3^ in size, were loaded into membrane-driven DACs with culet sizes of 500 μm. Tungsten gaskets were preindented to 50 μm thickness, and then sample chambers 250 μm in diameter were drilled prior to loading the crystals. Ruby crystals were included in the sample chamber for use as a pressure gauge.

Equations of state (EoS) were fitted to the volume-pressure data using EosFit7-GUI^25^ whereby the EoS were constrained to second-order (*B*_0_′ = 4) Birch–Murnaghan equations.^26^ The validity of the EoS fits was checked via the associated *F*_E_ versus *f*_E_ plots.^27^

### 2.3 *Ab Initio* Density Functional Theory Calculations

The *ab initio* simulations were carried out within the framework of density functional theory, DFT,^28^ with the Vienna *ab initio* Simulation Package, VASP.^29,30^ The projector augmented-wave, PAW, and pseudopotentials^31,32^ were employed and the plane-wave kinetic cutoff was extended up to 540 eV to ensure highly converged results. The integrations over the Brillouin zone, BZ, were carried out with *k*-points special samplings (4 × 4 × 3 and 5 × 4 × 3 grids, for the low and high-pressure phases, respectively). The exchange-correlation energy was described by means of the generalized gradient approximation, GGA, with the Armiento and Mattsson, AM05, prescription.^33,34^ To treat the strongly correlated states properly, the DFT+U method of Duradev et al.^35^ was employed. This method utilizes a single parameter, *U*_eff_ = *U* – *J*, where *U* and *J* are the effective on-site Coulomb and exchange parameters, respectively. The value used for *U*_eff_ was 6.5 eV for the Cu atoms.^36,37^ In the present study, the antiferromagnetic configuration was found to be the lower one in energy.

The unit cell parameters and the atomic positions were fully optimized to obtain, at selected volumes, the relaxed structure. The criteria imposed for the optimization were that the forces on the atoms were less than 0.003 eV/Å, and the deviations of the stress tensors from a diagonal hydrostatic form were lower than 0.1 GPa. In this way, the simulations provide a data set of volumes, energies, and pressures (from the stress tensor) that are fitted with a Birch–Murnaghan equation of state^26^ to obtain the theoretical equilibrium volume, the Bulk modulus, and the pressure derivatives.

The *k*-path for the electronic band structure calculations was chosen with the SeeK-path tool.^38^ The band structure analysis was carried out with the sumo package.^39^

## 3 Results and Discussion

### 3.1 Visual Observations

**Figure 1:**
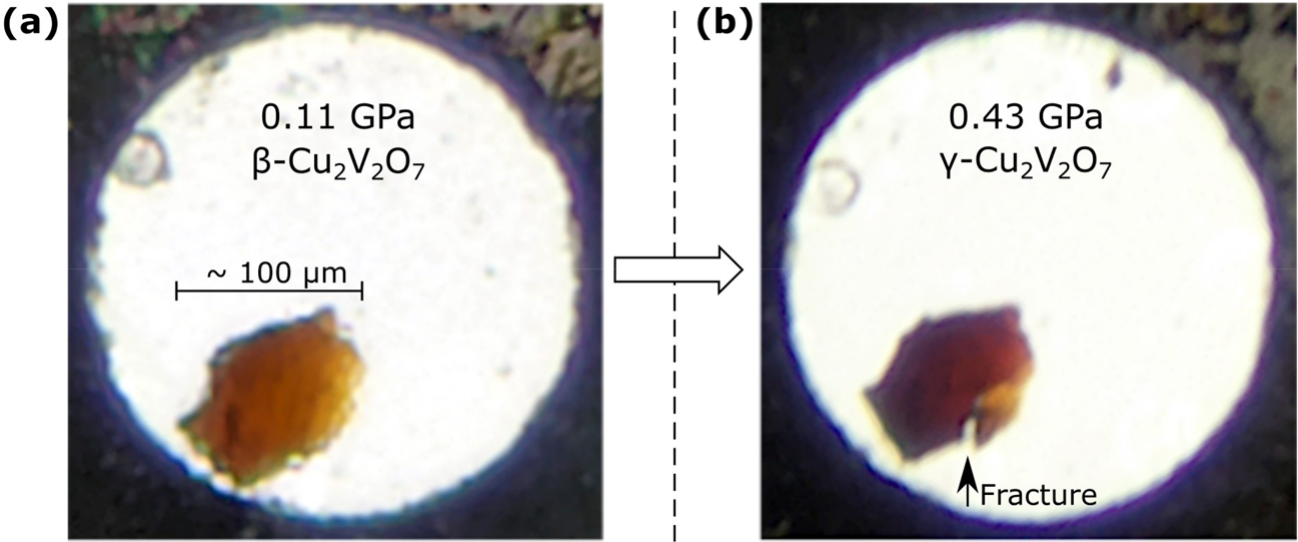
Micrographs of a Cu_2_V_2_O_7_ crystal (orange) in the sample chamber of a DAC. (a) β-Cu_2_V_2_O_7_ crystal at 0.11 GPa. (b) γ-Cu_2_V_2_O_7_ crystal at 0.43 GPa.

The pressure-induced β → γ phase transition is unambiguously confirmed by single-crystal XRD and *ab initio* density functional theory calculations (see the next section). Here, we begin by presenting two visual observations of the β → γ phase transition in the sample. First, the ∼2 eV band gap, which transmits light in the lower energy part of the visible spectrum (red/orange), is sensitive to pressure. In Figure 1 it is clear that the color of the Cu_2_V_2_O_7_ crystal becomes darker across the β → γ phase transition, which is associated with a band gap closure of ∼0.2 eV (see “Electronic Structure” section for further details). Second, as indicated by the arrow in Figure 1b, crystal fractures were observed on transition to the γ-phase. Neither crystal fracture nor color change necessarily indicates the existence of a phase transition; however, at such low pressures (<0.5 GPa), the fractures can indicate a large volume collapse associated with a first-order phase transitions. The transition is irreversible on sample decompression.

### 3.2 Structural Analysis and Phase Transition

**Figure 2:**
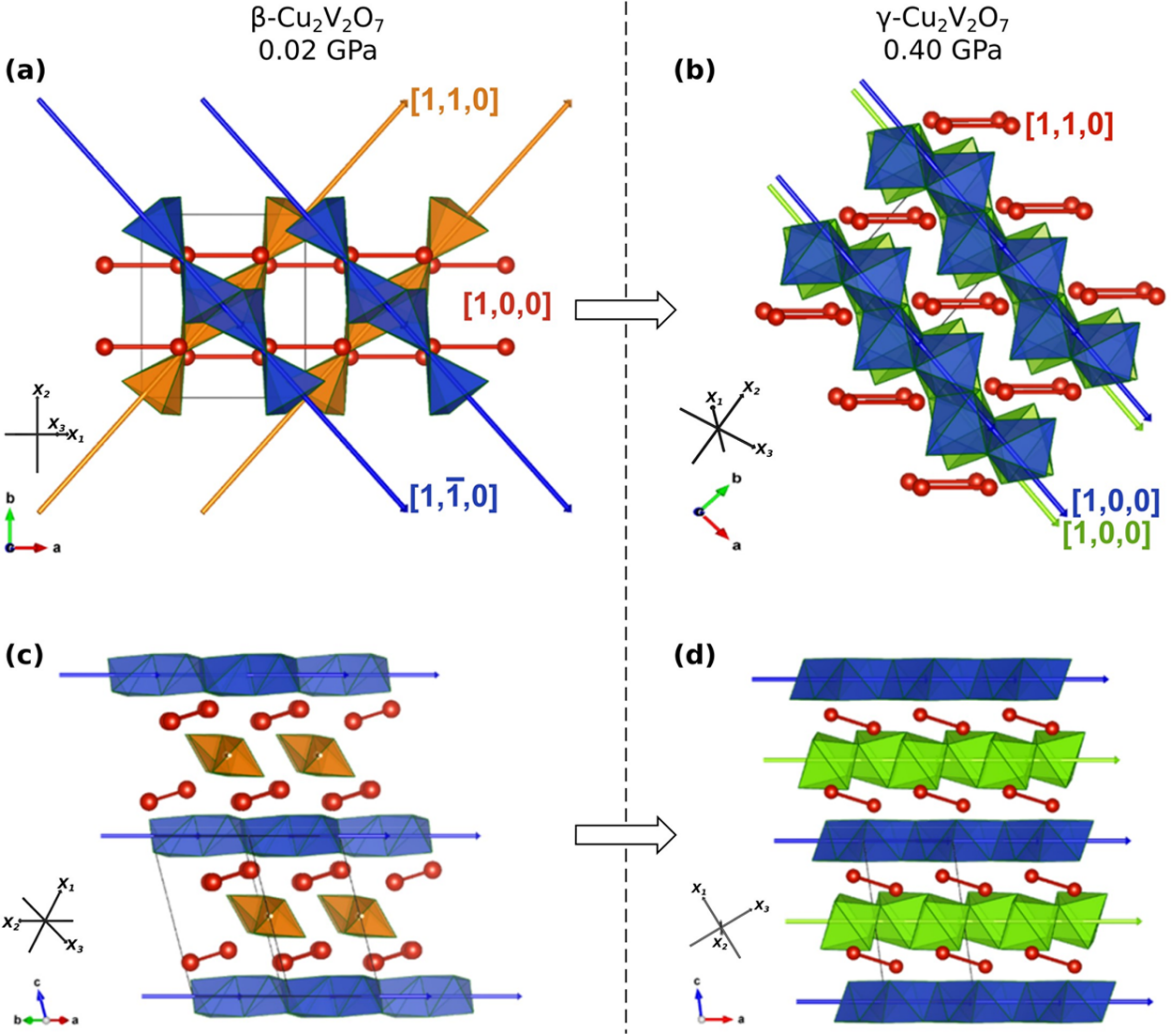
Crystal structures of β- and γ-Cu_2_V_2_O_7_. (a) and (b) β- and γ-Cu_2_V_2_O_7_ structures, respectively, projected along their *c*-axes, oriented so that the V–V vectors (red lines) of both structures are mutually parallel on the page. (c) and (d) β- and γ-Cu_2_V_2_O_7_ structures, respectively, projected along their *ab*-planes. Blue (and orange) polyhedra correspond to penta-coordinated CuO_5_ units. Green polyhedra correspond to hexa-coordinated CuO_6_ units. Red spheres represent vanadium atoms. For the sake of clarity, the VO_4_ tetrahedra and the oxygen atoms, which correspond to the vertices of all coordination complexes, are not shown. Blue, orange, and green vectors show the crystallographic direction of the chains. The black axes represent the principal compression axes (discussed in the compressibility section). The crystal structures were rendered in Vesta.^40^ Supplementary crystallographic data for both β- and γ-Cu_2_V_2_O_7_ structures can be obtained free of charge from the Cambridge Crystallographic Data Centre (CCDC) under deposition numbers 2123194–2123205.

The crystal structures of β- and γ-Cu_2_V_2_O_7_ are shown in Figure 2, and basic crystallographic information is presented in Table 1. Comprehensive crystallographic information is provided in Tables S1–S3. The crystal structures have previously been described elsewhere, determined from ambient pressure XRD measurements;^9^ however, for the sake of discussion, the structures of β- and γ-Cu_2_V_2_O_7_, as determined in the current work, are briefly described here.

**Table 1:** Basic Crystal Data for β- and γ-Cu_2_V_2_O_7_^a^

phase	β-Cu_2_V_2_O_7_		γ-Cu_2_V_2_O_7_	
data	experiment	DFT	experiment	DFT
pressure (GPa)	0.02	0.00	0.40	0.00
crystal system	monoclinic	monoclinic	triclinic	triclinic
space group	*C*2/*c*	*C*2/*c*	*P̅*1	*P̅*1
*a* (Å)	7.6858(11)	7.7660	5.080(5)	5.0540
*b* (Å)	8.0341(9)	7.9205	5.8098(16)	5.8041
*c* (Å)	10.121(3)	10.0631	9.380(4)	9.4689
α (deg)	90	90	100.00(3)	99.80
β (deg)	110.39(2)	109.36	97.20(6)	97.88
γ (deg)	90	90	97.18(5)	97.15
*V* (Å^3^)	585.8(2)	584.0	267.4(3)	268.0
*Z*	4	4	2	2

a Comprehensive experimental crystallographic information is provided in Tables S1–S3.

The structure of β-Cu_2_V_2_O_7_ is shown in Figures 2a,c. In β-Cu_2_V_2_O_7_, all of the Cu^2+^ cations are crystallographically equivalent, and all of them are penta-coordinated by oxygen atoms, thereby forming CuO_5_ coordination complexes with a square-based-pyramidal configuration. (For clarity, the CuO_5_ units are shown in blue and orange in Figure 2a,c.) All CuO_5_ units share edges to form continuous 1D chains which point along the *ab*-plane diagonals. The chains are also organized into layers, which follow the stacking sequence (ABAB...), where chains in layer A point in the [1,1,0] direction (orange), while chains in layer B point in the [1,®1,0] direction (blue). These A and B layers are interconnected by layers of V_2_O_7_ dimers, which are each formed by two corner-sharing VO_4_ tetrahedra. The V–V vectors, defined by the vanadium atoms in the V_2_O_7_ units, are all mutually parallel and point along the *a*-axis (i.e., in the [1,0,0] direction).

The structure of the γ-Cu_2_V_2_O_7_ phase is shown in Figure 2b,d. The γ-Cu_2_V_2_O_7_ structure has two symmetrically independent Cu^2+^ cations. Half of the Cu^2+^ cations are penta-coordinated in the same way as those in β-Cu_2_V_2_O_7_; however, the other half are hexa-coordinated, thereby forming CuO_6_ units with octahedral coordination configuration (shown in green in Figure 2b,d). All CuO_5_ (CuO_6_) units, share edges to form continuous 1D chains, shown in blue (green). All CuO_5_ and CuO_6_ chains point along the *a*-axis, or in the [1,0,0] direction. The chains are also organized into layers, which follow the stacking sequence (ABAB...), where chains in layer A contain only CuO_5_ units (blue), while chains in layer B contain only CuO_6_ units (green). These A and B layers are again interconnected by layers of V_2_O_7_ dimers. The V–V vectors are all mutually parallel and point along the *ab*-plane diagonal (i.e., in the [1,1,0] direction).

It is clear from Figure 2 that the crystal structures of β- and γ-Cu_2_V_2_O_7_ are closely related. Both structures are characterized by alternating layers of chains of CuO_*x*_ units, whereby the layers are interconnected by mutually parallel V_2_O_7_ units. The orientation of the V–V vectors (red) relative to the CuO_5_ chains (blue) is the same in both structures (shown in Figure 1). Therefore, the β → γ phase transition can be qualitatively described by two factors: (1) an increase of coordination number, from 5 to 6, of all CuO_5_ units in alternating layers and (2) a transformation of those layers (approximately by a reflection in the *ac*-plane) so that all CuO_6_ chains become parallel to the CuO_5_ chains.

Cu_2_V_2_O_7_ has not been studied previously under high-pressure conditions; therefore, this is the first time that the β → γ phase transition has been observed to be induced by pressure. Bearing in mind that β-Cu_2_V_2_O_7_ has been found to occur naturally in fumarloic areas and that the low pressure required to induce the β → γ phase transition (*<*0.4 GPa) occurs naturally in the earth’s crust, the present work shows that γ-Cu_2_V_2_O_7_ may also occur naturally although it has not yet been discovered. The β → γ phase transition has previously been observed to be induced by high temperatures (∼700 °C) at ambient pressures.^7,8^ Therefore, the β → γ phase boundary must have a steep negative slope in pressure–temperature space because it can be crossed by increasing either temperature (∼700 °C) or pressure (*<*0.4 GPa) from ambient conditions. *Ab initio* density functional theory (DFT) calculations of enthalpy, for the beta and gamma phases, found that the triclinic phase becomes the more stable phase at 0.1 GPa and 0 K (see Figure S1).

### 3.3 Electronic Structure

**Figure 3:**
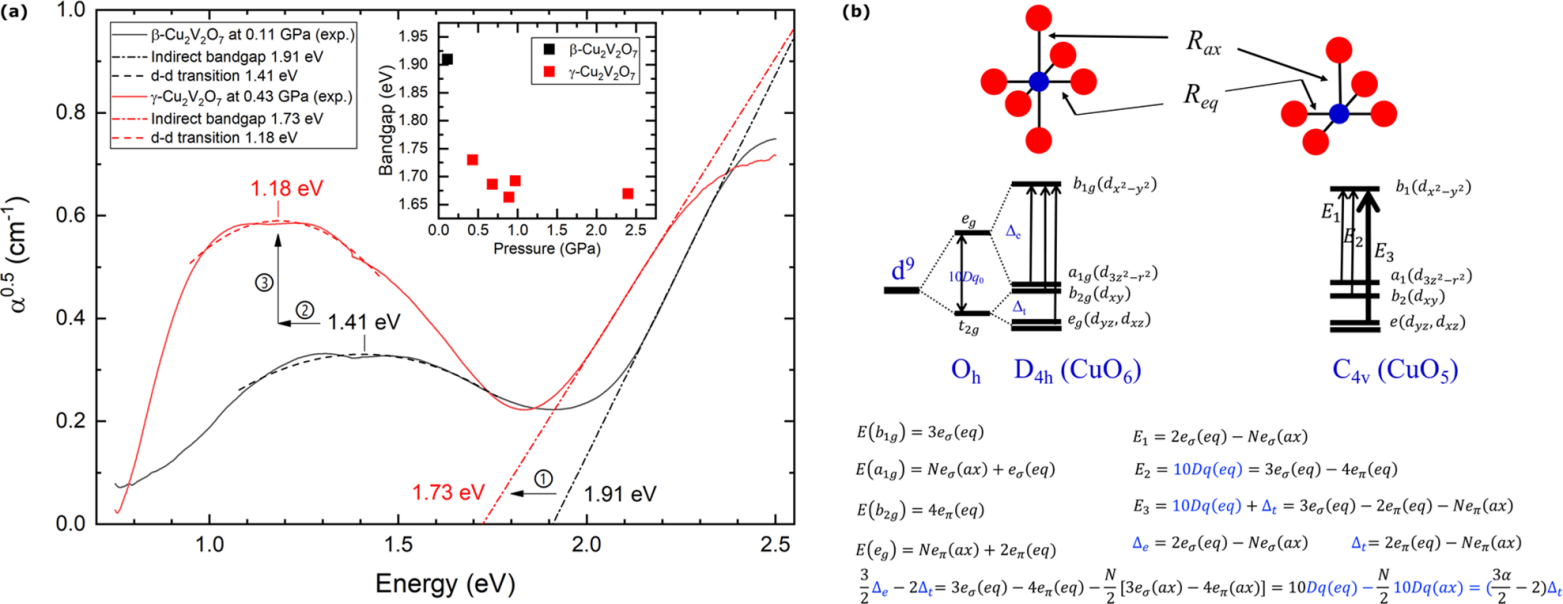
Absorption spectra and schematic energy level diagrams. (a) Experimental absorption spectra. Black lines correspond to β-Cu_2_V_2_O_7_ at 0.11 GPa. Red lines correspond to γ-Cu_2_V_2_O_7_ at 0.43 GPa. Dashed and dotted lines correspond to the linear fits determining the band gap energy. The band gap energies of β- and γ-Cu_2_V_2_O_7_ were experimentally determined to be 1.91 and 1.73 eV respectively. Dashed lines correspond to Gaussian fits determining the d-d transition energy. Inset: the experimental band gap as a function of pressure. The individual fitted spectra are shown in Figure S2. (b) Schematic energy level diagrams of the elongated octahedral CuO_6_ (nearly *D*_4*h*_) and elongated pyramidal CuO_5_ (nearly C_4*v*_) complexes.

The optical properties, and thus the piezo- and thermochromic properties, exhibited by β- and γ-Cu_2_V_2_O_7_ can be explained by the optical transmission window defined by the interband charge transfer transitions (optical band gaps) and the sub-band absorption of Cu^2+^ d-levels (see Figure 3). The relative variations in transition energies and oscillator strength (absorption coefficient) with temperature and/or pressure determine these properties. Therefore, understanding these properties requires a precise knowledge of how structural variations (determined via XRD) affect the electronic structure.

The electronic structures of β- and γ-Cu_2_V_2_O_7_ were investigated via absorption spectroscopy measurements and *ab initio* simulations. There are two regions of interest in the adsorption spectra in Figure 3: first, on the right-hand side (above 1.8 eV), the optical band gap edges; second, on the left-hand side (below 1.8 eV), the absorption associated with d–d electronic transitions of Cu^2+^.

#### 3.3.1 Band Structure

**Figure 4:**
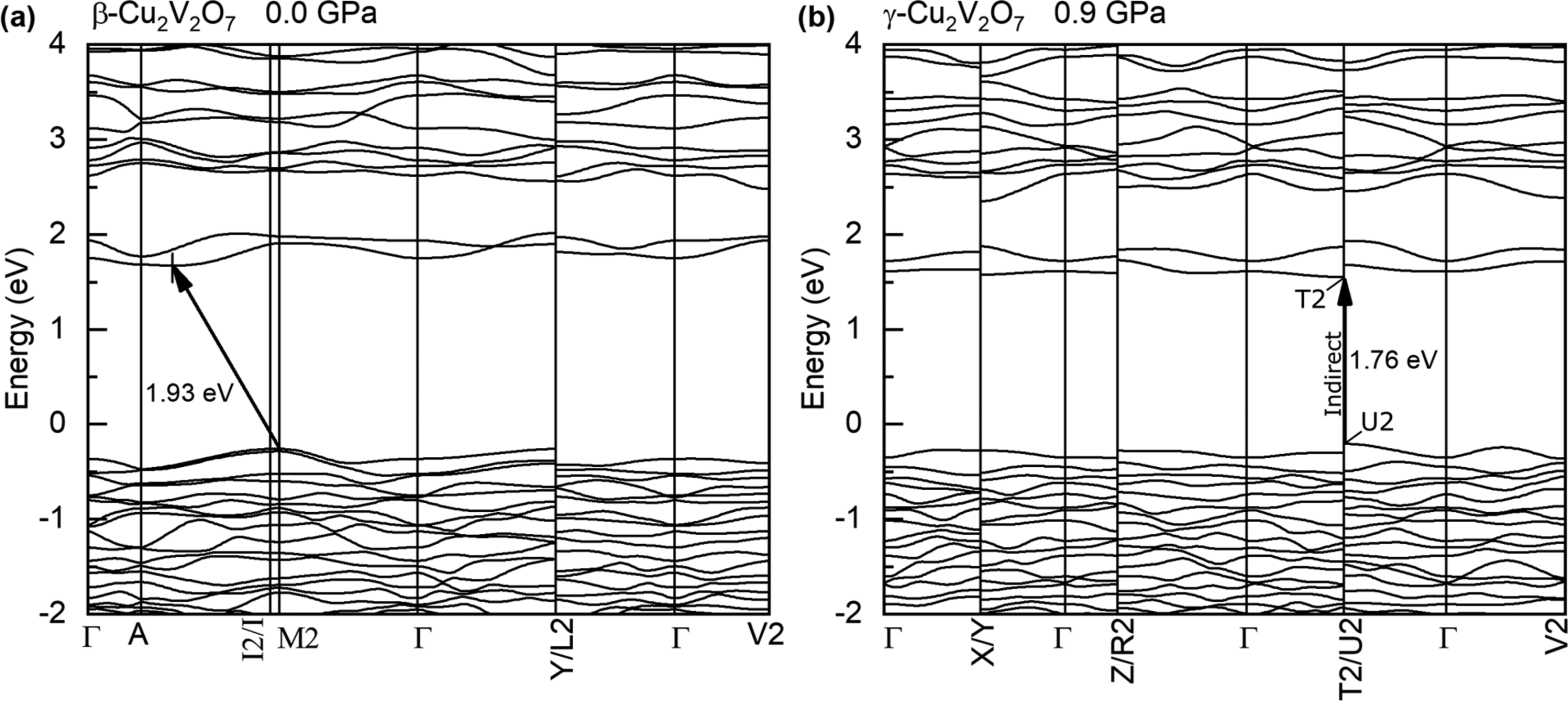
Calculated electronic band structures of β- and γ-Cu_2_V_2_O_7_. (a) β-Cu_2_V_2_O_7_ at 0.0 GPa. (b) γ-Cu_2_V_2_O_7_ at 0.9 GPa.

We first discuss the optical band gap energies. The band gap energies of β- and γ-Cu_2_V_2_O_7_ were experimentally determined to be 1.91 and 1.73 eV, respectively, thereby corresponding to a band gap collapse of 0.18 eV (∼9%) across the β → γ phase transition (as shown by arrow 1 in Figure 3). The β-phase band gap energy of 1.91 eV is in good agreement with the previously reported experimental values of 1.78 eV (ref (37)), 1.91 eV (ref (3)), and 2.21 eV (ref (41)) and the theoretically calculated value of 2.00 eV.^10^ The band gap of γ-Cu_2_V_2_O_7_ has not previously been reported. Density functional calculations were also carried out to obtain the full electronic band structures, as indicated in Figure 4, revealing both band gaps to be indirect. The calculated (experimental) band gap energies of β- and γ-Cu_2_V_2_O_7_ are 1.93 eV (1.91 eV) and 1.76 eV (1.73 eV) respectively, thereby showing excellent agreement between experiment and theory. The pressure evolution of the calculated band gaps up to 10 GPa is shown in Figure S3. The experimental band gaps as a function of pressure up to 2.4 GPa are shown in the inset in Figure 3. Additionally, the DOS (total density of states) and the projected density of states (Figure S4) are very similar for both phases. The upper level of the valence band is contributed to mainly by Cu-3d and O-2p states, while the O 2s states are located in the lower part of the valence band. As for the conduction band, the major contribution comes from the V 3d states.

#### 3.3.2 Sub-Band Absorption

We now discuss the sub-band absorption (below 1.8 eV) associated with d–d electronic transitions of Cu^2+^. The broad Gaussian-shaped absorbing regions observed in the sub-band gap energy region below 1.8 eV (see Figure 3) are due to d–d electronic transitions of Cu^2+^ and the transition energies can be thoroughly explained by crystal-field (CFM) and angular overlap (AOM) models.^42,43^ The maxima, bandwidth, and oscillator strength of these bands strongly depend on the Cu^2+^ local symmetry of the CuO_5_ or CuO_6_ coordination complexes.^44^Figure 3b shows schematic energy level diagrams of the elongated octahedral CuO_6_ (nearly *D*_4*h*_) and elongated pyramidal CuO_5_ (nearly *C*_4*v*_) complexes, and the corresponding energies as a function of the σ and π bonding AOM parameters *e*_σ_ and *e*_π_ for the equatorial (eq) and axial (ax) ligands.^42^ The crystal-field strength scales with the bond distance in oxides as a power of 5:10*Dq*(*R*) = *C*/(*R*^5^), where *R* is the bond length. This *R*-dependence is found from basic CF theory,^43,45,46^ and it has also been observed experimentally in several high-pressure experiments in transition metal oxides and fluorides involving Cr^3+^ (ref (47)), Fe^3+^ (refs (48−50)), or Co^2+^(ref (51)) within an experimental accuracy of 5.0 ± 0.5. Therefore, the ratio of the transition energies in the β- and γ-phases is given by
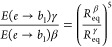
1The derivation of eq 1 is provided in the Supporting Information. Taking the average *R*_eq_^β^ = 1.95 Å and *R*_eq_^γ^ = 2.01 Å from the experimental XRD data, the reduction in *E*(*e* → *b*_1_) along the β → γ phase transition should be 0.86 according to the model. The experimental energy ratio between 1.41 eV (β) and 1.18 (γ) gives a ratio of 0.84 in excellent agreement with the model.

It must be noted that although *R*_eq_^β^ and *R*_eq_^γ^ are derived as an average of the four short Cu–O distances obtained from XRD for CuO_5_ the standard deviation of such distances is below σ = 0.012 Å in the β-phase and 0.07 Å in the γ-phase. In fact, the slight double-humped band observed in both phases (Figure 3a) may be due to low-symmetry local distortion of CuO_5_ beyond *C*_4*v*_. This symmetry lowering would additionally split the doubly degenerate *e* levels into two singlets (*xz*, *yz*) yielding additional broadened bands.

In Figure 3, both sub-band absorption spectra are completely determined by the CuO_5_ polyhedra because d–d transitions are, in the first approximation, parity-forbidden in centrosymmetric compounds like CuO_6_ (Laporte rule), although they can occur via electron–phonon coupling, thereby contributing only weakly to the observed spectrum. An additional simplification is that the *e*(d_*xz*_,d_*yz*_) → *b*_1_(d_*x*^2^–*y*^2^_) electric-dipole transition, which is allowed in CuO_5_, has an oscillator strength which is an order of magnitude higher than any of the other d–d transitions in CuO_5_ (involving the *b*_2_(d_*xy*_) and *a*_1_(d_3*z*^2^–*r*^2^_) levels), or in the nearly *D*_4*h*_ elongated CuO_6_ (*e*_*g*_, *b*_2*g*_, *a*_1*g*_ → *b*_1_).^45,52^ Therefore, only one transition is needed to explain the sub-band absorption peaks.

The sub-band absorption maxima are found at 1.41 and 1.18 eV for β- and γ-Cu_2_V_2_O_7_, respectively, thereby corresponding to a decrease in the transition energy of approximately 0.23 eV across the phase transition (as indicated by arrow 2 in Figure 3). The transition energy (or d-level splitting) in CuO_5_ is determined by the strength of the crystal field, which is in turn determined by the Cu–O bond lengths. The decrease in transition energy across the phase transition implies a decrease in crystal field strength and therefore an increase in Cu–O bond length. This is consistent with the Cu–O bond lengths determined from our XRD data (see Figure S5). For example, the average CuO_5_ equatorial (axial) Cu–O distance increases from 1.95 (2.25) Å to 2.01 (2.33) Å across the phase transition. For comparison, these values are in agreement with those found in α-CuMoO_4_, wherein the *e* → *b*_1_ absorption energy is identified as 1.49 eV in CuO_5_ units which have an average equatorial Cu–O distance of 1.93 Å (ref (52)). A similar energy of 1.7 eV was also reported in YBa_2_Cu_3_O_*y*_ in ref (53).

The observed oscillator strength (absorption coefficient) largely depends on the orientation of the transition dipole, which in this case is in the basal plane of the CuO_5_ polyhedra, and the electric field vector of the incident light used in the experiment. Because the γ-phase has exactly half the number of CuO_5_ polyhedra of the β-phase, a drop in oscillator strength would be expected across the phase transition, however the opposite is observed (as shown by arrow 3 in Figure 3). This can be attributed to the spatial orientation of the single crystal sample relative to the incident light. It is not possible to comment on the crystallographic orientation in the absorption spectra because the XRD measurements were carried out independently on different single crystal samples.

### 3.4 Bulk Modulus

**Figure 5:**
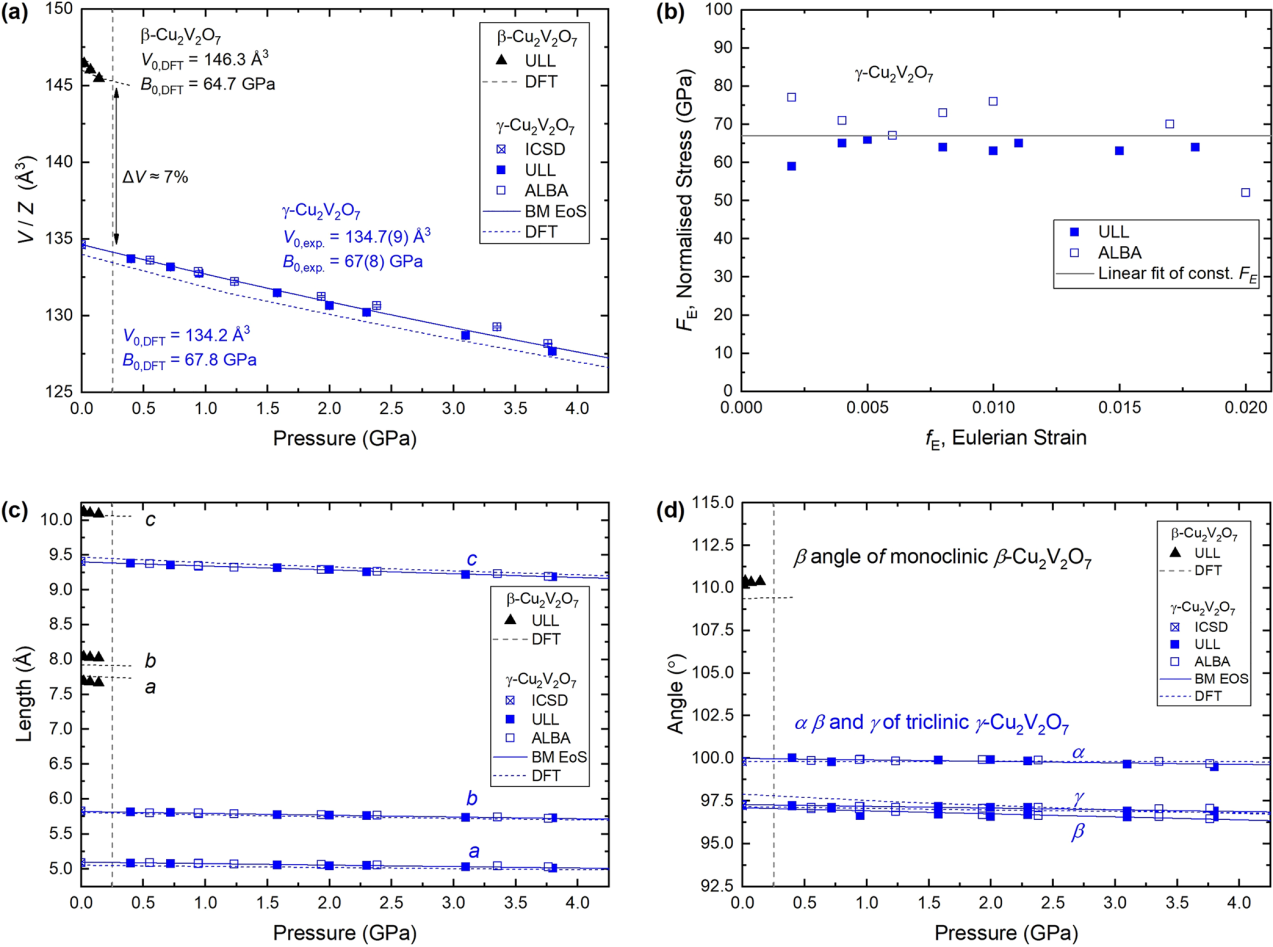
Equation of state and pressure-response of crystal lattice parameters in β- and γ-Cu_2_V_2_O_7_ to 4 GPa. (a) Normalized unit cell volume as a function of increasing pressure up to 4 GPa. (b) Normalized stress as a function of Eulerian strain for the experimental data. (c) Lattice constants *a*, *b*, and *c*, and (d) unit cell angles, α, β, and γ, as functions of pressure. Data corresponding to β-Cu_2_V_2_O_7_ (γ-Cu_2_V_2_O_7_) are shown in black (blue). (a, c, d) Solid (dashed) lines correspond to second-order Birch–Murnaghan equations of state (BM EoS) fitted to the experimental (calculated) data. Vertical dashed gray lines show the approximate phase transition pressure. The labels “ULL” and “ALBA” respectively signify data collected in-house at the University of La Laguna and at ALBA Synchrotron. ICSD refers to the ambient condition (atmospheric pressure and room temperature) γ-Cu_2_V_2_O_7_ structure of ref (9) taken from the Inorganic Crystal Structure Database.

The β → γ phase transition is unambiguously categorized as first-order according to the plot of the normalized unit cell volume (*V/Z*) as a function of pressure, as shown in Figure 5a. A volume collapse of approximately 7% is observed across the phase transition in both experimental and calculated data. The normalized ambient pressure volumes, *V*_0_, and bulk moduli, *B*_0_, were determined by fitting Birch–Murnaghan (BM) equations of state (EoS) truncated to second-order in energy (*B*_0_′ = 4) to the data. Due to the low phase transition pressure, only four experimental data points were obtained for β-Cu_2_V_2_O_7_. Therefore, the theoretical data (black dashed line in Figure 5a) are used for the discussion. The excellent reliability of the calculated data is clearly shown in Figure 5 (dashed lines) by the close agreement with the experimentally determined lattice parameters.

The normalized volume at ambient pressure, *V*_0_, and bulk modulus, *B*_0_, for both β- and γ-Cu_2_V_2_O_7_ (summarized in Figure 5a) are as follows: For the low-pressure phase, β-Cu_2_V_2_O_7_, the DFT data are fitted with *V*_0,DFT_ = 146.3 Å^3^ and *B*_0,DFT_ = 64.7 GPa. For the high-pressure phase, γ-Cu_2_V_2_O_7_, the parameters are *V*_0,DFT_ = 134.2(9) Å^3^ and *B*_0,DFT_ = 67.8 GPa, which are in excellent agreement with those determined from the experimental data: *V*_0,exp_ = 134.7(9) Å^3^ and *B*_0,exp._ = 67(8) GPa. Numbers in parentheses are the estimated standard error in the least significant digit. These bulk moduli are comparable to some of the lowest observed in other vanadate materials, for example Cu_3_V_2_O_8_ and Zn_2_V_2_O_7_, at 52(2) and 58(9) GPa, respectively.^54,55^

Figure 5b shows the normalized stress, *F*_E_, as a function of Eulerian strain, *f*_E_. The *F*_E_ versus *f*_E_ plot provides assessment of the quality of the fitted EoS, whereby a zero-gradient fit to the data indicates that a second-order truncation of the BM EOS is suitable (see ref (27) for more details). as is the case for γ-Cu_2_V_2_O_7_ (see Figure 5b).

### 3.5 Isothermal Compressibility

The isothermal compressibility tensor describes the principal axes of compression which, for any crystal system, constitute a unique set of orthogonal axes along which compressibility is described by a linear function of pressure.^57^ The magnitudes (compressibilities, *K*) and directions of the principal axes of compression (*X*_1_, *X*_2_, and *X*_3_) for β- and γ-Cu_2_V_2_O_7_ are presented in Table 2.

**Table 2:** Compressibility Data for β- and γ-Cu_2_V_2_O_7_^a^

β-Cu_2_V_2_O_7_			
axis	compressibility, *K* (GPa^–1^)	direction [*u*, *v*, *w*]	approx. direction [*x*, *y*, *z*]
*X*_1_	31.8 × 10^–3^	0.8031, 0.0000, 0.5959	4, 0, 3
*X*_2_	5.2 × 10^–3^	0.0000, 1.0000, 0.0000	0, 1, 0
*X*_3_	32.1 × 10^–3^	0.7818, 0.0000, −0.6236	4, 0, −3

γ-Cu_2_V_2_O_7_			
axis	compressibility, *K* (GPa^–1^)	direction [*u*, *v*, *w*]	approx. direction [*x*, *y*, *z*]
*X*_1_	5.75 × 10^–3^	–0.6089, 0.3500, 0.7118	–12, 7, 14
*X*_2_	4.59 × 10^–3^	–0.0136, 0.9924, −0.1222	0, 1, 0
*X*_3_	2.28 × 10^–3^	0.9262, 0.2496, 0.2826	4, 1, 1

a The principal compression axes (*X*_1_, *X*_2_, and *X*_3_) are represented by black arrows in Figures 2 and 6. The data in the table were calculated using the lattice parameters from the single crystal XRD analysis (Tables S1–S3) and the PASCal principal axis strain calculator.^56^.

The principal axes of compression of β-Cu_2_V_2_O_7_ reveal a pronounced anisotropic compressibility (see Figures 2 and 6) which is herein rationalized in terms of the underlying crystal structure. The principal axis of minimal compression, in this case *X*_2_, points in the [0,1,0] direction and has a compressibility of 5.2 × 10^–3^ GPa^–1^. Therefore, the least compressible direction in β-Cu_2_V_2_O_7_ exactly corresponds to the crystallographic *b*-axis and points exactly between the layers of chains of edge-sharing CuO_5_ units (which lie in the *ab*-plane). Additionally, *X*_2_ dissects the smallest angle between the two different types of chains (see Figure 2a) thereby pinpointing the direction of maximum strength.

**Figure 6:**
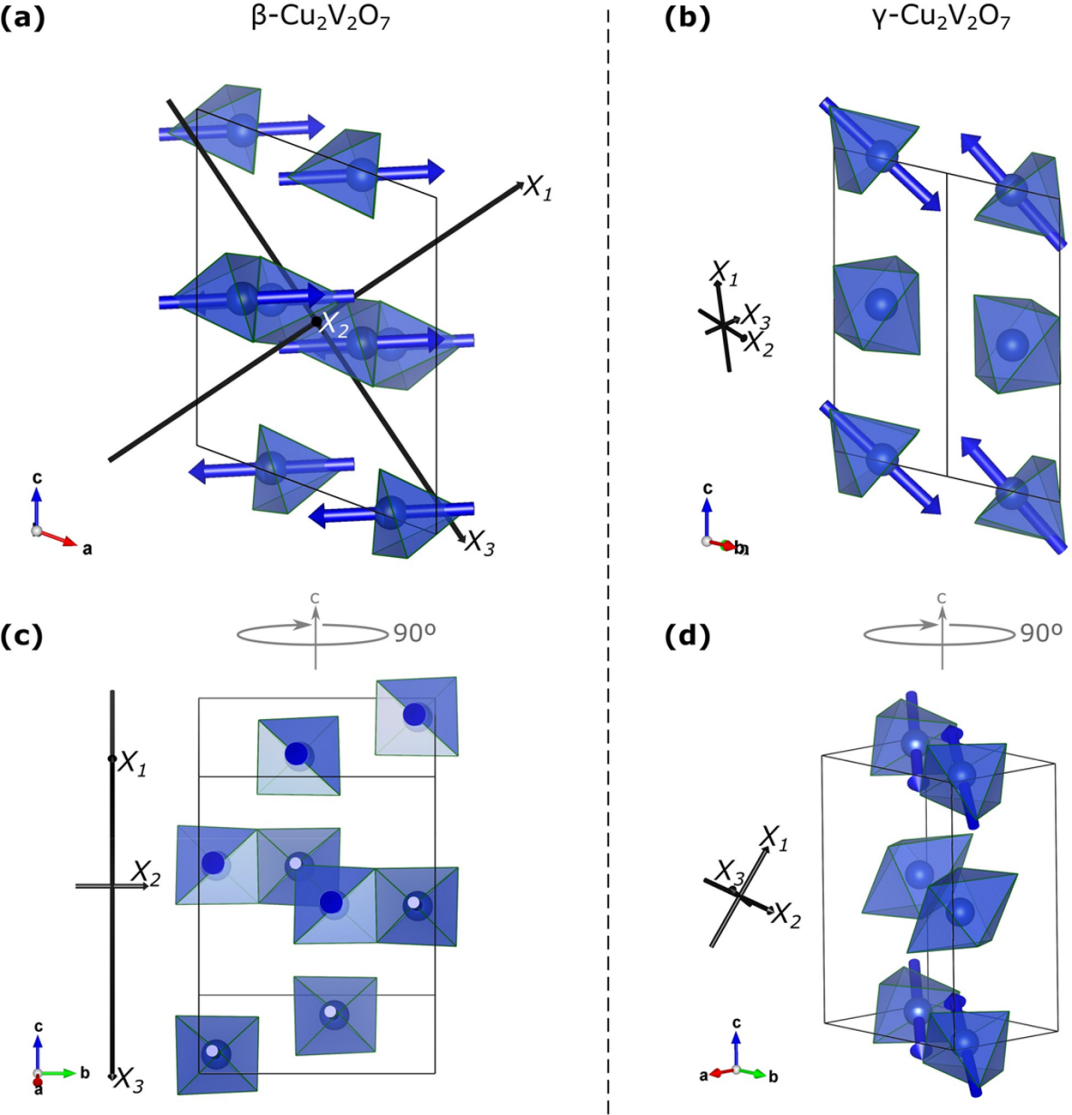
Crystal structures of β- and γ-Cu_2_V_2_O_7_ showing the lone electron pair (LEP) vectors. (a, c) β-Cu_2_V_2_O_7_ and (b, d) γ-Cu_2_V_2_O_7_. Blue arrows show the direction of the LEP on each CuO_5_ unit (blue polyhedra). Black arrows represent the compressibility axes. The arrow length shown in the figure is proportional to the magnitude of compressibility for the corresponding axis.

In contrast, the intermediate and major principal compression axes (*X*_1_ and *X*_3_) in β-Cu_2_V_2_O_7_ are approximately 6 times more compressible than *X*_2_, with mutually comparable compressibilities of 31.8 × 10^–3^ and 32.1 × 10^–3^ GPa^–1^ respectively. Both *X*_1_ and *X*_3_ lie in *ac*-plane, and as per the definition of the principal axes of compression, they are related by a rotation of 90° since their directions are [4,0,3] and [4,0,–3], respectively. It is important that both of *X*_1_ and *X*_3_ lie in the *ac-*plane because they share this description with all of the CuO_5_ lone electron pairs (LEPs) (see Figure 6a,c). Specifically, each penta-coordinated CuO_5_ units possesses an axial LEP, opposite the axial oxygen atom, which points toward the location where a sixth oxygen would make a complete octahedron. The directions of the LEPs are indicted by blue arrows in Figure 6. The LEPs are exactly perpendicular to the principal axis of minimal compression, *X*_2_ (equivalently, the *b*-axis); therefore, it is clear that the LEPs are responsible for the highly anisotropic compressibility of β-Cu_2_V_2_O_7_. A similar phenomenon has also been observed in iodates.^58^

The principal axes of compression of γ-Cu_2_V_2_O_7_ reveal a much less pronounced anisotropic compressibility in contrast to that of β-Cu_2_V_2_O_7_. Where the principal axes of major and minor compression in the β-phase respectively had compressibilities of 32.1 × 10^–3^ and 5.2 × 10^–3^ GPa^–1^ (a difference of a factor of approximately 6), in the γ-phase, they have compressibilities of 5.75 × 10^–3^ and 2.28 × 10^–3^ GPa^–1^ (a difference of a factor of approximately 2). Interestingly, the most compressible axis in the γ-phase phase is roughly equivalent to the least compressible axis in the β-phase in terms of compressibility.

### 3.6 Pressure-Induced Structural Evolution/Relation to Nonlinear Thermal Expansion

The β-Cu_2_V_2_O_7_ structure exhibits negative thermal expansion (NTE) at ambient pressures. The primary mechanism for the NTE is believed to relate primarily to a transverse vibrational mode associated with the oxygen atom which bridges the VO_4_ tetrahedra in the V_2_O_7_ units (O_3_V–O–VO_3_). The V_2_O_7_ units interlink the chains of edge-sharing CuO_*x*_ units; therefore, a vibrational mode perpendicular to the V–O–V bridge causes a reduction in the time-averaged distance between the vanadium atoms, thereby pulling the layers closer together and reducing the unit volume.^14,15^

**Figure 7:**
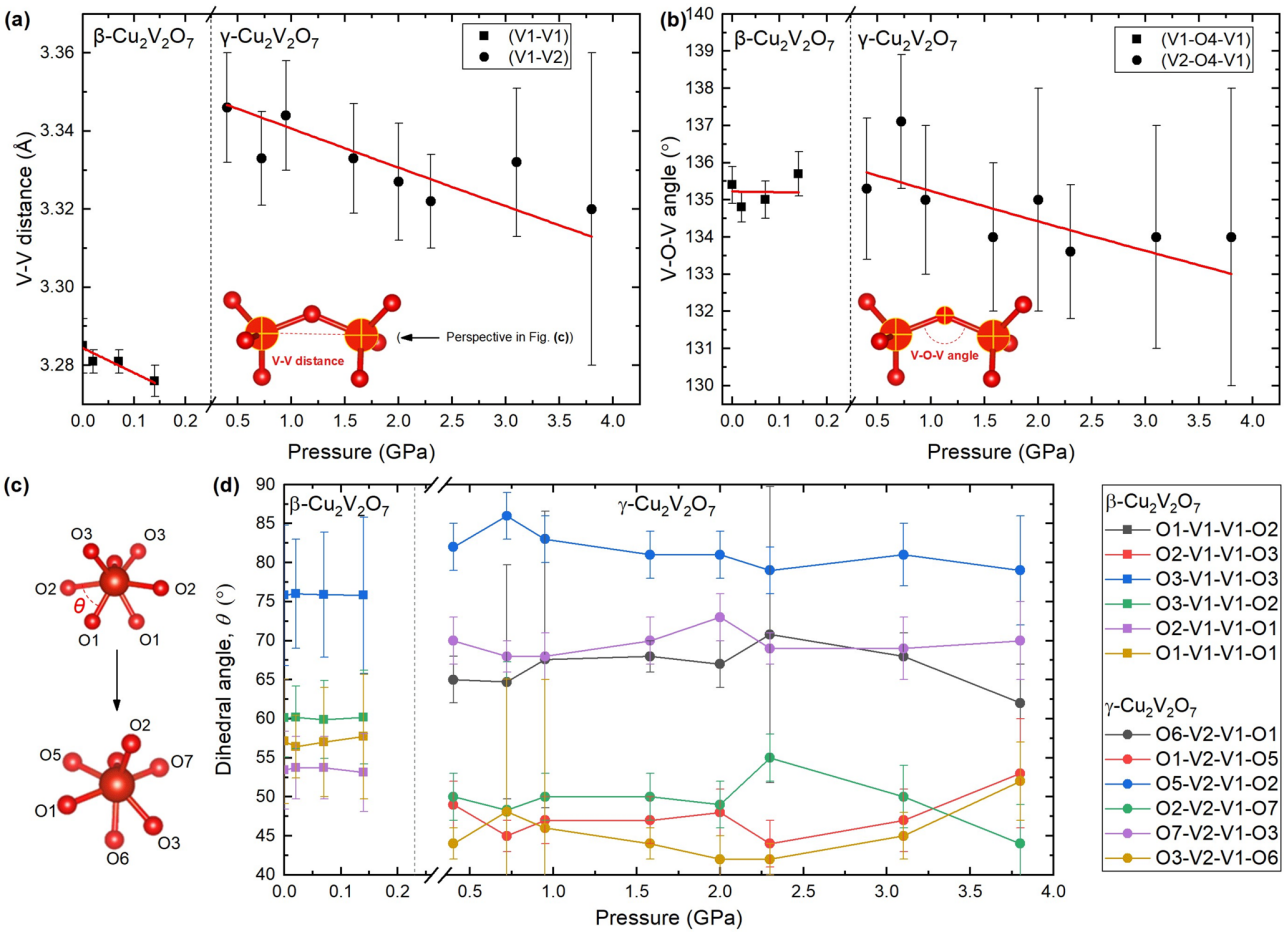
Pressure evolution of the V_2_O_7_ dimers in β- and γ-Cu_2_V_2_O_7_. (a) Interatomic distance between vanadium atoms. (b) Angle between the V–O bonds, forming the V–O–V angle. (c) V_2_O_7_ molecules viewed along the axis defined by the vanadium atoms before (top) and after (bottom) the phase transition. (d) Pressure evolution of the O–V–(O)–V–O dihedral angles, θ.

Since the layers lie in the *ab*-plane, a reduction in the lattice constant *c* corresponds to a decrease in the interlayer distance. As shown in Figure 5c, all lattice constants for both phases decrease monotonically with increasing pressure. In the present work, the interlayer spacing abruptly decreases from 4.7429 to 4.5294 Å across the β → γ phase transition, thereby corresponding to a change in interlayer spacing of −0.2135 Å. Because only the time-averaged atomic positions can be observed in XRD measurements, the vibrational modes cannot be observed directly. However, the time-averaged V_2_O_7_ unit provides insight regarding the pressure-induced structural behavior. For example, Figure 7a shows the intervanadium (V–V) distance as a function of pressure. The V–V distance decreases with pressure in both phases; however, it jumps by +0.1 Å across the β → γ phase transition. The normalized unit cell volume decreases across the transition, therefore the V–V distance is clearly not the dominating factor in the volume decrease. Additionally, as shown in Figure 7b, the V–O–V angle, formed by the bridging oxygen atom, appears to remain constant in the β-phase, whereas it decreases with pressure in the γ-phase and exhibits no clear jump across the phase transition. Therefore, the V–O–V angle is also not responsible for the large volume collapse. The Cu–O bond distances also remain roughly constant over the full pressure range (see Figure S5), and in fact, the volume of the CuO_*x*_ coordination complexes appears to increase (see Figure S6). The key to the volume collapse of Δ*V* ≈ 7% therefore likely lies with the mutual rotation of the VO_4_ units. According to the XRD data, when viewed along the V–V direction (as shown in Figure 7c) the O_3_V–O–VO_3_ configuration goes from staggered in the β-phase to slightly eclipsed in the γ-phase. In a perfectly staggered configuration all of the O–V–(O)–V–O dihedral angles, θ, would be equal to 60°. All V_2_O_7_ dihedral angles are plotted as a function of pressure in Figure 7d, whereby it is clear that the dihedral angles in the γ-phase exhibit a larger deviation from 60° than those in the β-phase. This is important because it suggests that the relative rotation of the VO_4_ units (which comprise the V_2_O_7_ dimers) is an important characteristic of the volume collapse. This supports previous ambient pressure investigations which suggest that mutual rotation of the VO_4_ molecular units, suggested by vibrational spectroscopy measurements, underpin the negative thermal expansion mechanism.^14,15^

## 4 Conclusions

We report an experimental high-pressure single-crystal synchrotron XRD study of Cu_2_V_2_O_7_ under compression to 4 GPa at ambient temperature. The results unambiguously reveal a previously unknown first-order pressure-induced phase transition from monoclinic β-Cu_2_V_2_O_7_ to triclinic γ-Cu_2_V_2_O_7_ below 0.40 GPa. The β → γ phase transition is associated with a volume collapse of approximately 7%, an increase in oxygen coordination (from 5 → 6) of half of the Cu^**2+**^ coordination complexes, and a reorientation of 1-dimensional chains composed of edge sharing CuO_6_ units. The phase transition is also characterized by an indirect → indirect electronic band gap decrease of approximately 0.2 eV as measured by absorption spectroscopy (1.93 → 1.75 eV) and calculated via density functional calculations (1.93 → 1.76 eV). The band gap of γ-Cu_2_V_2_O_7_ has been measured here for the first time, indicating that γ-Cu_2_V_2_O_7_ has better potential as a photocatalyst for water splitting than does β-Cu_2_V_2_O_7_ due to its lower band gap and therefore greater absorption of visible light. The pressure evolution of the crystal lattice parameters and isothermal compressibility tensor are also reported here for the first time. The compressibility of β-Cu_2_V_2_O_7_ is found to be highly anisotropic, with the axis of minimal compression being approximately six times less compressible than the other two axes. The anisotropic compressibility is rationalized in terms of the underlying crystal structures and the presence and spatial orientation of lone-electron pairs on the constituent CuO_5_ units. Due to the low transition pressure at ambient temperature, large volume collapse, and the fact that Cu_2_V_2_O_7_ is a multiferroic material, this combined experimental and theoretical investigation into the high-pressure structural and electronic evolution of Cu_2_V_2_O_7_ suggests that in addition to the previously suggested uses in photocatalytic water splitting Cu_2_V_2_O_7_ could also be a very relevant material for exploring solid state barocaloric effects, such as solid-state refrigeration technologies based on crystals which are environmentally friendly. For a material to be promising in terms of barocaloric effects it needs to exhibit, among other qualities, a first-order like phase transition involving large structural changes (e.g., volume change) near room temperature induced by small pressure drifts (i.e., of the order of 0.1 GPa). As shown in the current work, Cu_2_V_2_O_7_ appears to meet these specifications.

## Supporting Information Available

Detailed crystallographic tables; calculated relative enthalpy; absorption spectra; calculated band gap energies; density of states and projected density of states; derivation of the ratio of sub-band transition energies; pressure evolution of the Cu–O bonds in CuO_5_ and CuO_6_ polyhedra; and CuO_*x*_ and VO_4_ polyhedral volumes as functions of pressure (PDF)

## Accession Codes

CCDC 2123194–2123205 contain the supplementary crystallographic data for this paper. These data can be obtained free of charge via www.ccdc.cam.ac.uk/data_request/cif, or by emailing data_request@ccdc.cam.ac.uk, or by contacting The Cambridge Crystallographic Data Centre, 12 Union Road, Cambridge CB2 1EZ, UK; fax: +44 1223 336033.
