# Supplementary material for: Pressure-Induced Phase Transition and Band Gap Decrease in Semiconducting β-Cu2V2O7
Source: Inorg Chem. 2022 Feb 14;61(8):3697–707. doi: 10.1021/acs.inorgchem.1c03878 (PMC8889581; doi:10.1021/acs.inorgchem.1c03878)
Supplement: Supplementary file 1 — ic1c03878_si_001.pdf [file ic1c03878_si_001.pdf]

Supporting Information: Pressure-induced phase transition and band-gap decrease in semiconducting $\beta-Cu_2V_2O_7$

Robin Turnbull[a,*], Javier González-Platas[b], Fernando Rodríguez[c], Akun Liang[a], Catalin Popescu[d], Zhangzhen He[e], David Santamaría-Pérez[a], Plácida Rodríguez-Hernández[f], Alfonso Muñoz[f] and Daniel Errandonea[a]

[a] Departamento de Física Aplicada - Instituto de Ciencia de Materiales, MALTA Consolider Team, Universidad de Valencia, Edificio de Investigación, C/Dr. Moliner 50, Burjassot, 46100 Valencia, Spain.

[b] Departamento de Física - Instituto Universitario de Estudios Avanzados en Física Atómica, Molecular y Fotónica (IUDEA), MALTA Consolider Team, Universidad de La Laguna, Avenida Astrofísico Fco. Sánchez s/n, La Laguna, Tenerife E-38204, Spain.

[c] MALTA Consolider Team, Departamento de Ciencias de la Tierra y Física de la Materia Condensada, Facultad de Ciencias, Universidad de Cantabria, 39005 Santander, Spain.

[d] CELLS-ALBA Synchrotron Light Facility, 08290 Cerdanyola del Vallès, Barcelona, Spain.

[e] State Key Laboratory of Structural Chemistry, Fujian Institute of Research on the Structure of Matter, Chinese Academy of Sciences, Fuzhou, Fujian 350002, China.

[f] Departamento de Física, Instituto de Materiales y Nanotecnología, MALTA Consolider Team, Universidad de La Laguna, La Laguna, E-38204 Tenerife, Spain.

∗e-mail: robin.turnbull@uv.es

Supplementary Table S1 | Details of the data collection, refinement results, and structural data obtained from *β*-$Cu_2V_2O_7$ at different pressures.

**Pressure (GPa)**	**0.00**	**0.02**	**0.07**	**0.14**
Formula	$Cu_2V_2O_7$	$Cu_2V_2O_7$	$Cu_2V_2O_7$	$Cu_2V_2O_7$
$D_{calc.}$/ g cm^{-3}	3.866	3.866	3.877	3.892
μ /mm^{-1}	10.233	10.234	10.264	10.303
Formula Weight	340.96	340.96	340.96	340.96
Colour	brown	brown	brown	brown
Shape	plate	plate	plate	plate
Size/mm^3	0.11×0.08×0.05	0.09×0.07×0.05	0.09×0.07×0.05	0.09×0.07×0.05
T/K	298(1)	298(1)	298(1)	298(1)
Crystal System	monoclinic	monoclinic	monoclinic	monoclinic
Space Group	*C 2/c*	*C 2/c*	*C 2/c*	*C 2/c*
a/Å	7.682(5)	7.6858 (11)	7.6761(10)	7.6645 (18)
b/Å	8.0399(9)	8.0341 (9)	8.0304(8)	8.0272(14)
c/Å	10.112 (5)	10.121 (3)	10.104(3)	10.089 (4)
α/°	90	90	90	90
β/°	110.27(7)	110.39(2)	110.31(2)	110.38(4)
γ/°	90	90	90	90
V/Å^3	585.8 (5)	585.8(2)	584.1(2)	581.9(3)
Z	4	4	4	4
Wavelength/Å	0.71073	0.71073	0.71073	0.71073
Radiation type	MoKα	MoKα	MoKα	MoKα
$2\theta_{min}$/°	7.7	7.60	7.60	7.61
$2\theta_{max}$/°	56.32	56.44	56.52	56.34
Measured Refl.	414	777	736	533
Independent Refl.	226	306	295	279
R_{int}	0.0112	0.0146	0.0214	0.0309
Parameters	35	35	35	35
Restraints	0	0	0	0
Largest Peak	0.40	0.85	0.71	0.58
Deepest Hole	-0.55	-0.76	-0.51	-0.61
GooF	1.112	1.106	1.065	1.100
wR_2 (all data)	0.0780	0.0841	0.0889	0.1033
wR_2	0.0763	0.0813	0.0863	0.0934
R_1 (all data)	0.0335	0.0385	0.0415	0.0539
R_1	0.0292	0.0331	0.0363	0.0415

Supplementary Table S2 | Details of the data collection, refinement results, and structural data obtained from γ-$Cu_2V_2O_7$ at different pressures.

**Pressure (GPa)**	**0.40**	**0.72**	**0.95**	**1.58**
Formula	$Cu_2V_2O_7$	$Cu_2V_2O_7$	$Cu_2V_2O_7$	$Cu_2V_2O_7$
$D_{calc.}$/ g cm^{-3}	4.235	4.252	4.265	4.307
μ /mm^{-1}	11.210	11.257	11.291	11.401
Formula Weight	340.96	340.96	340.96	340.96
Colour	brown	brown	brown	brown
Shape	plate	plate	plate	plate
Size/mm^3	0.09×0.07×0.05	0.09×0.07×0.05	0.09×0.07×0.05	0.09×0.07×0.05
T/K	298(1)	298(1)	298(1)	298(1)
Crystal System	triclinic	triclinic	triclinic	triclinic
Space Group	*P -1*	*P -1*	*P -1*	*P -1*
a/Å	5.080(5)	5.0735(16)	5.081(3)	5.0575(18)
b/Å	5.8098(16)	5.802(4)	5.7838(10)	5.7686(15)
c/Å	9.380(4)	9.352(4)	9.336(2)	9.313(3)
α/°	100.00(3)	99.77(4)	99.916(10)	99.86(3)
β/°	97.20(6)	97.05(4)	96.63(3)	96.71(3)
γ/°	97.18(5)	97.10(4)	97.16(3)	97.16(3)
V/Å^3	267.4(3)	266.3(2)	265.47(19)	262.92(15)
Z	2	2	2	2
Wavelength/Å	0.71073	0.71073	0.71073	0.71073
Radiation type	MoKα	MoKα	MoKα	MoKα
$2\theta_{min}$/°	7.20	7.73	7.23	7.25
$2\theta_{max}$/°	49.43	49.17	52.64	52.67
Measured Refl.	739	879	1174	1131
Independent Refl.	251	248	283	279
R_{int}	0.0374	0.0189	0.0402	0.0426
Parameters	53	48	48	48
Restraints	0	0	0	0
Largest Peak	0.75	0.71	0.99	1.03
Deepest Hole	-0.61	-0.73	-0.65	-0.74
GooF	1.117	1.152	1.142	1.128
wR_2 (all data)	0.1214	0.1489	0.1365	0.1479
wR_2	0.1145	0.1391	0.1294	0.1410
R_1 (all data)	0.0582	0.0618	0.0639	0.0654
R_1	0.0481	0.0532	0.0555	0.0579

Supplementary Table S3 | Details of the data collection, refinement results, and structural data obtained from γ-$Cu_2V_2O_7$ at different pressures.

**Pressure (GPa)**	**2.00**	**2.30**	**3.10**	**3.80**
Formula	$Cu_2V_2O_7$	$Cu_2V_2O_7$	$Cu_2V_2O_7$	$Cu_2V_2O_7$
$D_{calc.}$/ g cm^{-3}	4.333	4.349	4.399	4.436
μ/mm^{-1}	11.471	11.511	11.646	11.742
Formula Weight	340.96	340.96	340.96	340.96
Colour	brown	brown	brown	brown
Shape	plate	plate	plate	plate
Size/mm^3	0.09×0.07×0.05	0.09×0.07×0.05	0.09×0.07×0.05	0.09×0.07×0.05
T/K	298(1)	298(1)	298(1)	298(1)
Crystal System	triclinic	triclinic	triclinic	triclinic
Space Group	*P -1*	*P -1*	*P -1*	*P -1*
a/Å	5.044(5)	5.0487(19)	5.025(5)	5.006(7)
b/Å	5.7620(16)	5.7556(15)	5.7328(19)	5.723(4)
c/Å	9.287(4)	9.256(4)	9.215(4)	9.184(6)
α/°	99.90(3)	99.82(3)	99.62(4)	99.47(6)
β/°	96.56(5)	96.66(3)	96.55(5)	96.54 (8)
γ /°	97.10(4)	97.10(3)	96.88(5)	96.87 (8)
V/Å^3	261.3(3)	260.39(16)	257.4(3)	255.3 (4)
Z	2	2	2	2
Wavelength/Å	0.71073	0.71073	0.71073	0.71073
Radiation type	MoKα	MoKα	MoKα	MoKα
$2\theta_{min}$/°	7.26	7.26	7.28	7.29
$2\theta_{max}$/°	52.64	52.70	52.68	46.33
Measured Refl.	1152	1410	1084	789
Independent Refl.	274	363	275	210
R_{int}	0.0574	0.0189	0.0660	0.1486
Parameters	48	53	53	48
Restraints	0	0	0	0
Largest Peak	0.86	1.33	0.94	0.72
Deepest Hole	-0.69	-1.06	-0.82	-0.84
GooF	1.125	1.112	1.115	1.104
wR_2 (all data)	0.1439	0.1593	0.1447	0.2193
wR_2	0.1270	0.1385	0.1293	0.1915
R_1 (all data)	0.0733	0.0852	0.0878	0.1182
R_1	0.0574	0.0671	0.0642	0.0784

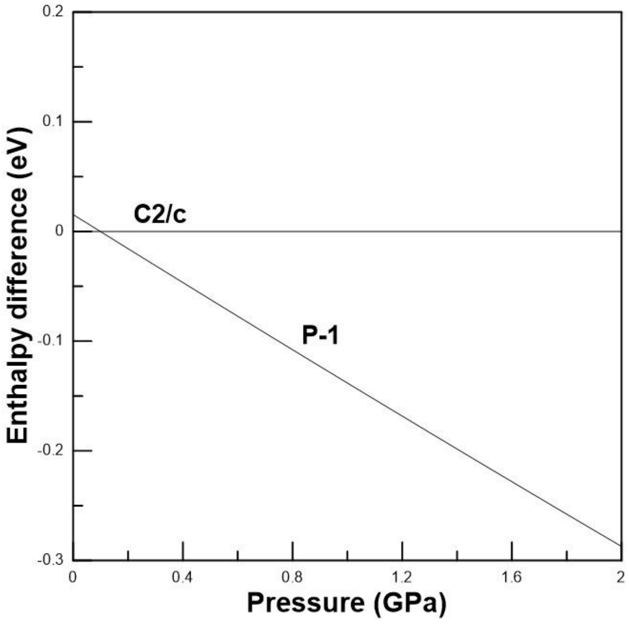

Supplementary Figure S1 | Calculated enthalpy for the monoclinic (*C*2/*c*) β−$Cu_2V_2O_7$ and triclinic (*P*−1) γ−$Cu_2V_2O_7$ phases. The enthalpy of the monoclinic phase is taken as reference. The triclinic phase becomes the more stable phase at 0.1 GPa.

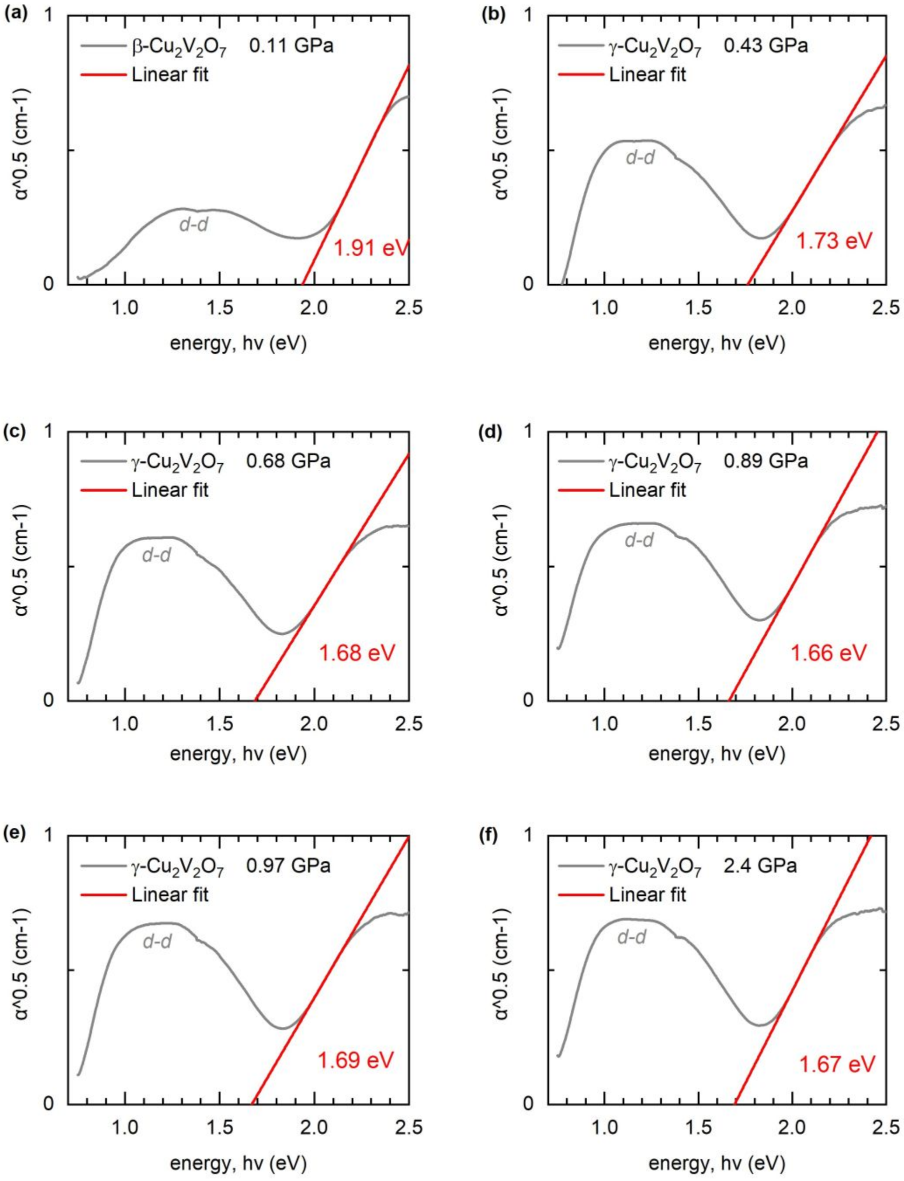

Supplementary Figure S2 | Absorption spectra from $Cu_2V_2O_7$ up to 2.4 GPa. (a) $\beta-Cu_2V_2O_7$. **(b-f)** $\gamma-Cu_2V_2O_7$. The slight dip in intensity at 1.4 eV in all spectra is due to the fact that the displayed data were acquired using two different spectrometers with different energy ranges. 1.4 eV corresponds to the cut-off energy between the two spectrometers.

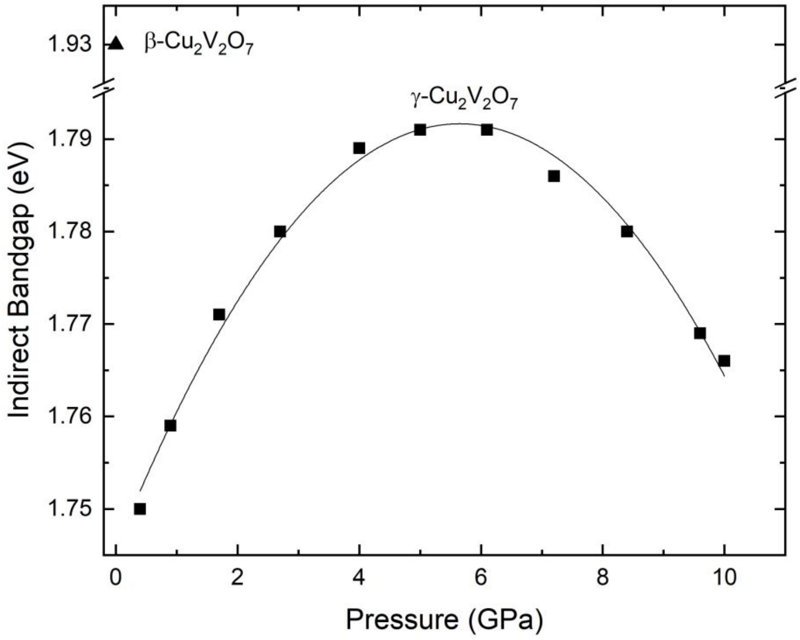

Supplementary Figure S3 | Calculated indirect bandgap energies of *β*− and *γ*−$Cu_2V_2O_7$.

The data are fitted with a parabolic curve.

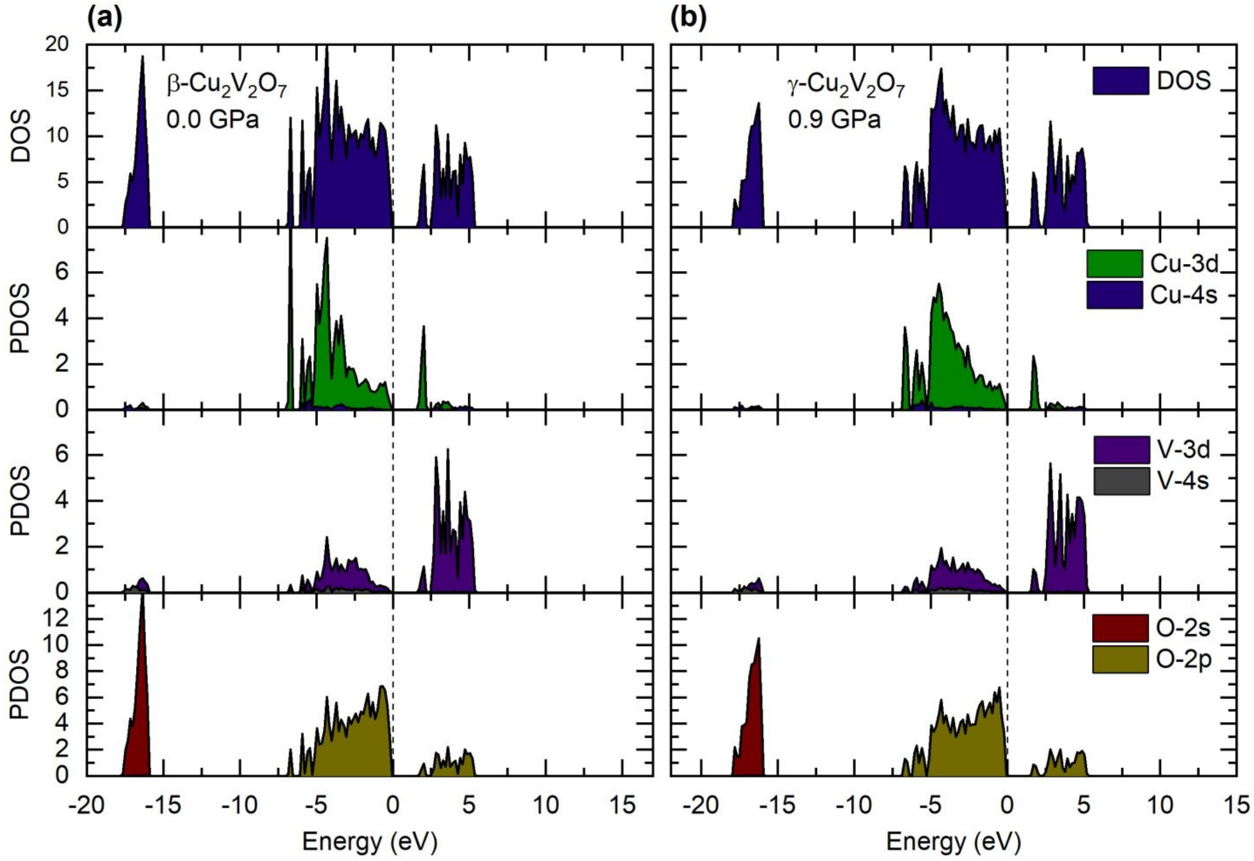

Supplementary Figure S4 | Density of states (DOS) and projected density of states (PDOS) for *β*− and *γ*−$Cu_2V_2O_7$. (a) *β*−$Cu_2V_2O_7$ at 0.0 GPa. **(b)** *γ*−$Cu_2V_2O_7$ at 0.9 GPa. The vertical dashed line is a guide to the eye to identify the top of the valence band.

Derivation of ratio of d-d transition energies in $\beta-$ and $\gamma-Cu_2V_2O_7$: $\frac{E(e\rightarrow b_1)\gamma}{E(e\rightarrow b_1)\beta} = (\frac{R_{eq}}{R_{ax}})^5$

The relationship between the octahedral crystal field 10Dq and the AOM parameters is given by the equation: $10Dq = 3e_\sigma - 4e_\pi$ (Refs. 42,43,45,46). As indicated in Fig. 3b, the transition energy $t_{2g} \rightarrow e_g$ in O_h, $b_{2g} \rightarrow b_{1g}$ in D_{4h} ($b_2 \rightarrow b_1 in C_{4v}$) corresponds to $10Dq$ for an octahedral and equatorial (short Cu-O) bond distance, respectively. In this model, the energy of the electric-dipole allowed transition in CuO_5 is given by:

$$\begin{aligned} E(e \rightarrow b1) &= 10Dq(eq) + \Delta_t \\ &= 3e_\sigma(eq) - 2e_\pi(eq) - e_\pi(ax) \end{aligned} \tag{1}$$

following the scheme of Fig. 3b. $\Delta_t = 2e_\pi(eq) - e_\pi(ax)$ is the axial field splitting of the parent octahedral t_{2g} levels. The relationship between these AOM expressions and R_{ax} and R_{eq} can be carried out through $10Dq$ using the following relationship:

$$\begin{aligned} \frac{3}{2}\Delta_e - 2\Delta_t &= 3e_\sigma(eq) - 4e_\pi(eq) - \frac{1}{2}\left[3e_\sigma(ax) - 2e_\pi(ax)\right] \\ &= 10Dq(eq) - \frac{1}{2}10Dq(ax) \\ &= (\frac{3\alpha}{2} - 2)\Delta_t \end{aligned} \tag{2}$$

where α is the ratio $\Delta_e : \Delta_t$. This expression allows us to express the axial splitting Δ_t as a function of the CF parameters for the R_{ax} and R_{eq} bond distances as:

$$\begin{aligned} \Delta_t &= \frac{[10Dq(eq) - \frac{1}{2}10Dq(ax)]}{\frac{3\alpha}{2} - 2} \\ &= \frac{10Dq(eq)}{\frac{3\alpha}{2} - 2}\left[1 - \frac{1}{2}(\frac{R_{eq}}{R_{ax}})^5\right] \end{aligned} \tag{3}$$

Using the relationship established between Δ_e and Δ_t established in Ref. 44, we obtain that $\Delta_e = \alpha\Delta_t$, with $\alpha \approx 4$, in $\beta{-}Cu_2V_2O_7$. Therefore, taking (1) and (3) we obtain the relationship for the $e \rightarrow b_1$ transition energy as:

$$\begin{aligned} E(e \rightarrow b_1) &= 10Dq(eq) + \Delta_t \\ &= 10Dq(eq)\left[1.5 - 0.125(\frac{R_{eq}}{R_{ax}})^5\right] \\ &= 1.44 \times 10Dq(eq) \end{aligned} \quad (4)$$

This expression is valid for both phases: $\beta{-}Cu_2V_2O_7$ at 0.11 GPa (R_{ax} = 2.25 Å and R_{eq} = 1.95 Å) and $\gamma{-}Cu_2V_2O_7$ at 0.43 GPa (R_{ax} = 2.33 Å and R_{eq} = 2.01 Å), since the ratio $(\frac{R_{eq}}{R_{ax}})^5$ is 0.49 and 0.48 respectively. Therefore, the ratio of the transition energies in the $\beta-$ and $\gamma-$phases is given by:

$$\frac{E(e \rightarrow b_1)\gamma}{E(e \rightarrow b_1)\beta} = \left(\frac{R_{eq}^{\beta}}{R_{eq}^{\gamma}}\right)^5 \quad (5)$$

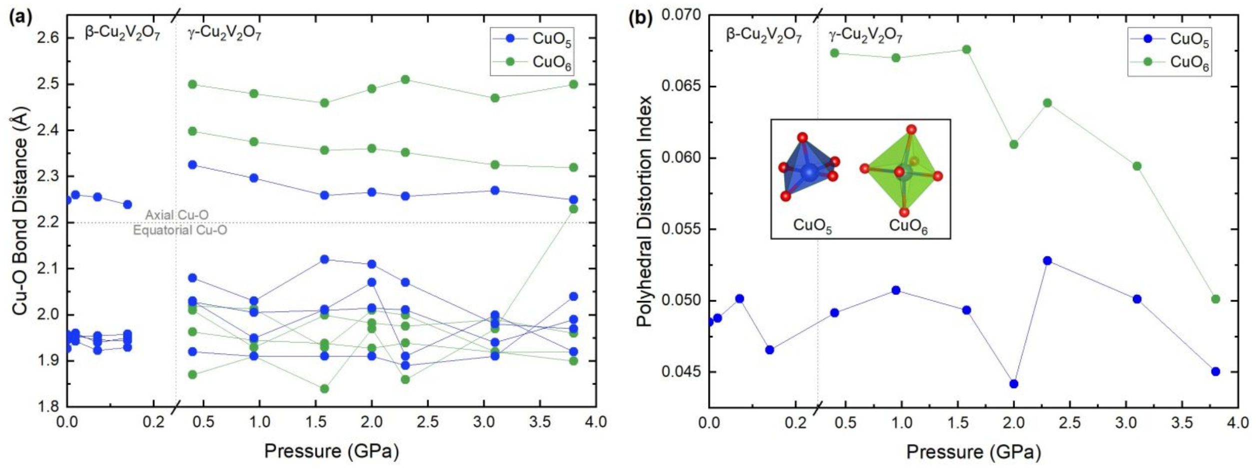

Supplementary Figure S5 | Pressure evolution of the Cu-O bonds and of the CuO_5 and CuO_6 polyhedra in $\beta-$ and $\gamma-Cu_2V_2O_7$. (a) Cu-O bond distances and **(b)** polyhedral distortion index as functions of pressure. Inset: CuO_5 and CuO_6 polyhedra.

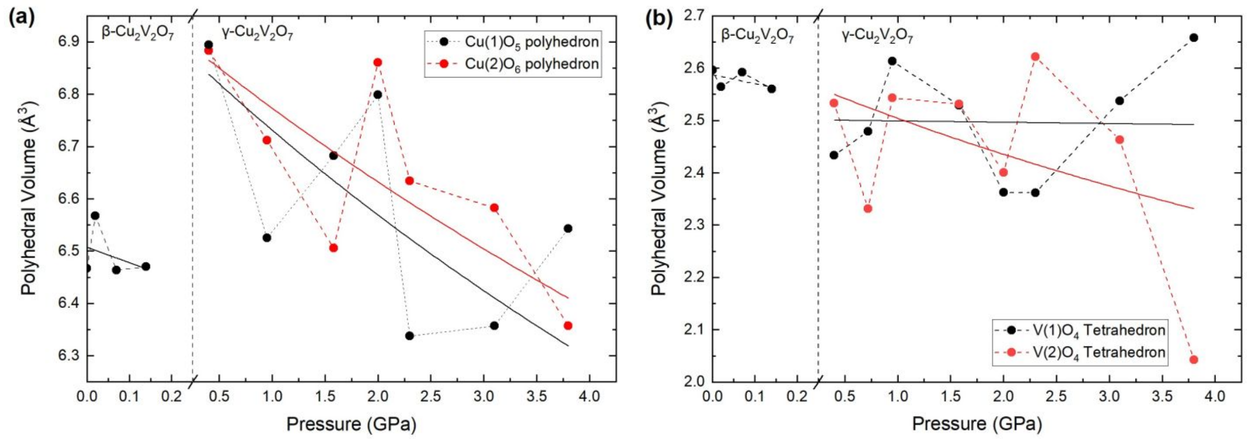

Supplementary Figure S6 | Coordination complex volumes as functions of pressure. (a) Cu-O coordination complexes. **(b)** VO_4 tetrahedral units.
